# Mycotic Aneurysm Due to Burkholderia pseudomallei: A Case Report From Malaysia

**DOI:** 10.7759/cureus.77177

**Published:** 2025-01-09

**Authors:** Chen Yi Cham, Katarina Shin Yee Choo, Giri Shan Rajahram, Chee Yik Chang

**Affiliations:** 1 Internal Medicine, Hospital Queen Elizabeth II, Kota Kinabalu, MYS; 2 Infectious Diseases, Hospital Queen Elizabeth II, Kota Kinabalu, MYS; 3 Infectious Diseases, Hospital Sultanah Aminah, Johor Bahru, MYS

**Keywords:** burkholderia pseudomallei, melioidosis, mycotic aortic aneurysm, saccular aneurysm, vascular infection

## Abstract

Melioidosis is a potentially life-threatening infectious disease caused by the Gram-negative bacterium *Burkholderia pseudomallei*. Its clinical manifestations are highly diverse, including pneumonia, abscesses in internal organs, non-healing ulcers, bone and joint infections, and encephalomyelitis. Mycotic aneurysms, a rare but serious complication of melioidosis, arise as sequelae of bacteremia involving the arterial wall. Despite appropriate antimicrobial therapy and surgical intervention, the mortality rate associated with this complication remains significant. In this report, we present a case of a melioidosis-related mycotic aneurysm of the distal aorta, successfully managed with a combination of targeted antimicrobial therapy and surgical intervention. This case highlights the challenges in diagnosing and treating such a rare manifestation of melioidosis.

## Introduction

Melioidosis is an infection caused by the Gram-negative bacterium *Burkholderia pseudomallei*, endemic in Southeast Asia and northern Australia. Melioidosis can manifest with various clinical presentations, including pneumonia, skin, and soft tissue infection, genitourinary infection, visceral abscesses, ocular melioidosis, neurological melioidosis, and septic arthritis [1]. Melioidosis is associated with a high mortality rate, particularly when presenting as an acute, fulminant illness. It primarily affects individuals with regular contact with soil and water, and risk factors include diabetes mellitus, hazardous alcohol use, pre-existing renal disease, immunosuppressive therapy, and thalassemia [2]. The most common routes of *B. pseudomallei* infection are believed to be inoculation, inhalation, and ingestion. Person-to-person transmission of *B. pseudomallei* is extremely rare, with sexual transmission suggested but not definitively proven [3].

The Darwin Prospective Melioidosis Study found that pneumonia was the most common presentation, found in half of the patients with culture-confirmed melioidosis, followed by skin infection, genitourinary infection, bacteremia with no evident focus, and soft tissue abscesses [4]. A melioidosis-related mycotic aneurysm is uncommon, but it is well recognized as potentially life-threatening, with a high morbidity and mortality rate. Here, we present a case of a melioidosis-related mycotic aneurysm involving the distal aorta, which was successfully treated with a combination of targeted antimicrobial therapy and surgical intervention.

## Case presentation

**Figure 1:**
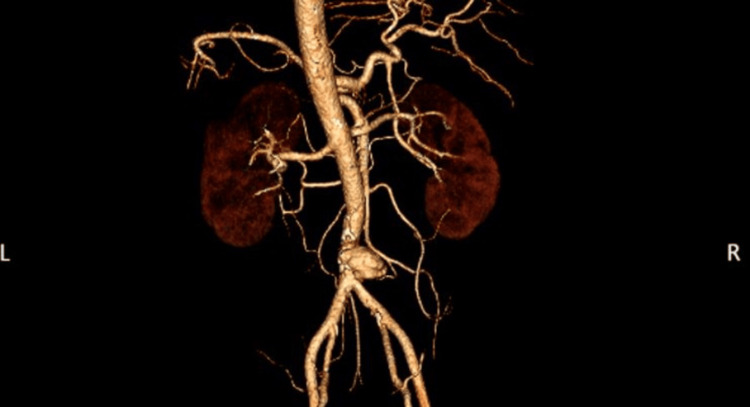
CT angiography of the abdomen showing a saccular aneurysm over the distal infrarenal abdominal aorta, measuring approximately 2.1 x 2.1 x 2.0 cm

**Figure 2:**
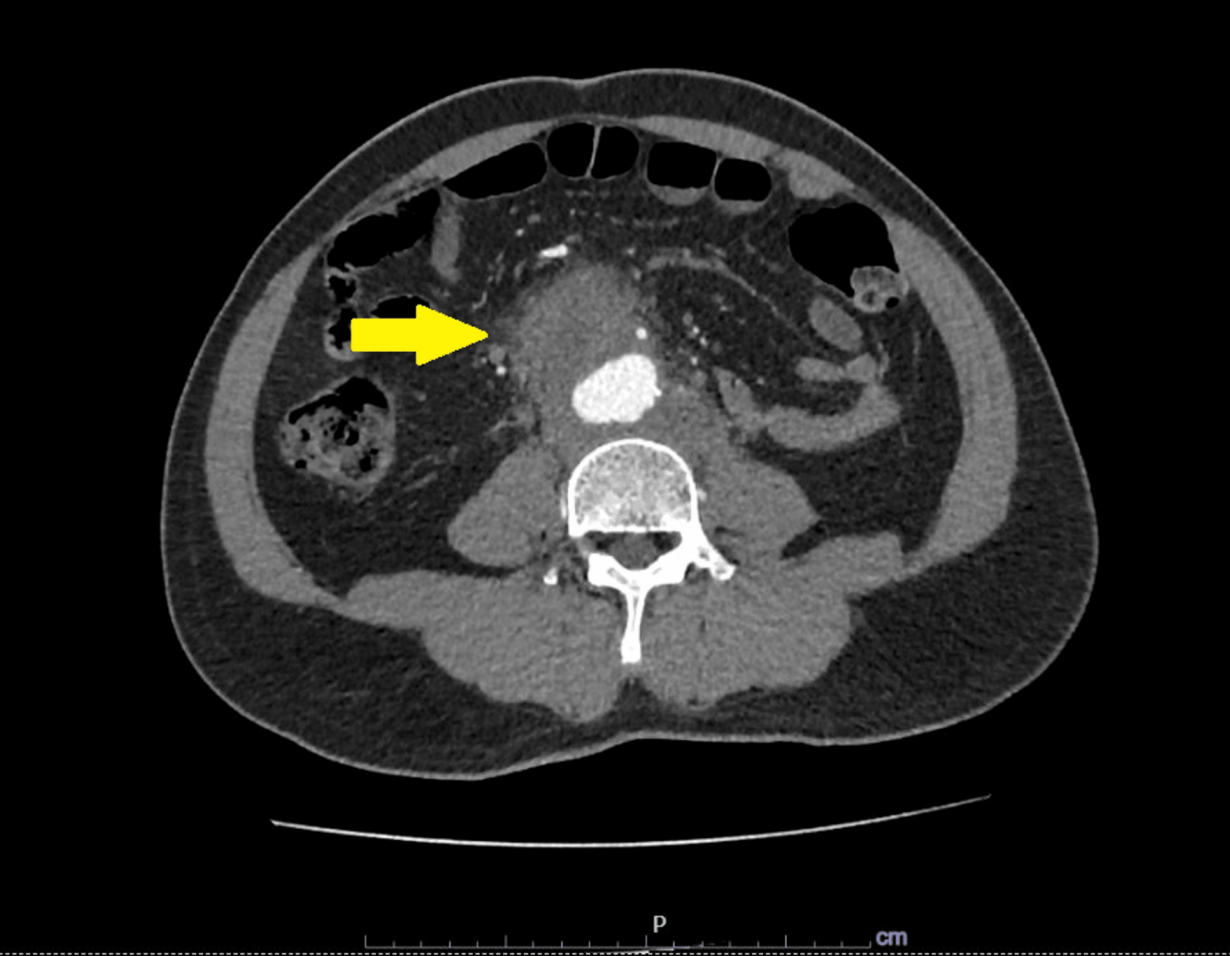
Axial view of the CT scan of the abdomen showing the presence of surrounding perianeurysmal hematoma extending anterolaterally into the right retroperitoneal region, measuring approximately 5.6 x 4.5 x 6.8 cm

A 51-year-old man with newly diagnosed type 2 diabetes mellitus presented with a four-week history of fever, lower back pain, and reduced appetite. He works as a technician at a ship repair dock. Initially, he sought care at a district hospital, where he was treated symptomatically and discharged with oral analgesics (NSAIDs) for lower back pain. As his symptoms worsened, he visited a private hospital two weeks later. A contrast-enhanced computed tomography (CT) scan of the thorax, abdomen, and pelvis revealed a saccular aneurysm at the distal aorta (Figure 1 and Figure 2). He was subsequently referred to our center for further management.

On admission, the patient was hemodynamically stable and afebrile, with an oxygen saturation of 98% while breathing ambient air. The cardiac examination revealed no heart murmurs, and the abdomen was soft with tenderness detected at the lower abdomen. The remainder of the physical examination was unremarkable. Initial blood investigations revealed leukocytosis with a white cell count of 14.3 × 10³/μL (reference range: 4-10 × 10³/μL), hemoglobin of 14.4 g/dL (reference range: 13-17 g/dL), platelets of 313 × 10³/μL (reference range: 150-410 × 10³/μL), and an elevated C-reactive protein (CRP) of 149.2 mg/L (reference range: <5 mg/L). A recent HbA1c was 10%, indicative of poorly controlled diabetes mellitus. He was given intravenous ceftazidime 2 grams every eight hours as an empirical antibiotic.

The patient was closely monitored in the cardiothoracic unit, and the antibiotic ceftazidime was continued. Despite receiving the antibiotic, his abdominal pain did not improve. On day 5 of admission, the patient developed severe abdominal pain, necessitating an emergency laparotomy. Intraoperatively, a tear was identified on the right lateral surface of the aneurysm, accompanied by slough and turbid fluid. The procedure involved open repair, thorough washing, and graft placement. The blood culture was negative, but intraoperative tissue and pus cultures grew *B. pseudomallei*, which was sensitive to trimethoprim-sulfamethoxazole, ceftazidime, doxycycline, and meropenem. Consequently, the diagnosis of a melioidosis-related mycotic aneurysm was established.

Post-operatively, the patient was started on intravenous meropenem. He required low-dose inotropic support due to septic shock and was managed in the intensive care unit (ICU). During his ICU stay, he also developed other complications, including anuric acute kidney injury necessitating intermittent hemodialysis and bilateral acute lower limb ischemia due to graft thrombosis. These complications were successfully managed with bilateral femoral embolectomy and revision of the distal aortic graft anastomosis.

The patient responded well to treatment, with improvement in septic parameters, including normalization of the white cell count and a decrease in CRP levels. Antibiotic therapy was switched to intravenous ceftazidime, which was completed over eight weeks. However, the patient experienced vomiting episodes during a graded transition to oral trimethoprim-sulfamethoxazole. He was subsequently planned for lifelong maintenance therapy with oral doxycycline. The patient’s condition stabilized, and he was discharged home.

## Discussion

Melioidosis is caused by the Gram-negative bacillus *B. pseudomallei*, which is endemic to Southeast Asia and Australia. It can affect any organ in the body and presents with a wide range of clinical manifestations, including pneumonia, genitourinary infections, skin and soft tissue infections, internal organ abscesses, septic arthritis, neurological or ocular melioidosis, and fulminant septicemia without an evident focus [5]. Significant predisposing risk factors for melioidosis include diabetes mellitus, preexisting renal diseases, thalassemia, and occupational exposure to contaminated soil and water [2].

In Southeast Asian countries, the most common causative pathogens for mycotic aneurysms are non-typhoidal Salmonella species and Staphylococcus aureus; thus, melioidosis-related mycotic aneurysms are exceedingly rare [6]. Melioidosis-related mycotic aneurysm accounts for only 1-2% of melioidosis cases but is associated with high rates of morbidity, mortality, and relapse. *B. pseudomallei* has been identified as the most common causative agent of mycotic aneurysms in northeastern Thailand, accounting for 17 out of 40 cases (42.5%) [7]. In contrast, a melioidosis-related mycotic aneurysm is less frequent in the Darwin melioidosis cohort, diagnosed in only two out of 540 patients (0.4%) [8]. Apart from that, there are three cases of melioidosis-related mycotic aneurysms reported in Sabah, Malaysia [9].

Mycotic aneurysms can involve different segments of the aorta, with the abdominal aorta being the most frequently affected. Wu et al. reported that, among 159 cases of bacteremic melioidosis, eight patients had mycotic aneurysms. Of these, six involved the abdominal aorta, one had a left iliac aneurysm, and another had an infectious mesenteric aneurysm [10]. *B. pseudomallei* bacteremia can result in hematogenous seeding of preexisting atherosclerotic vessels, leading to mycotic aneurysms. The pathogen’s virulence factors degrade the arterial wall, causing aneurysmal dilation with a risk of rupture and life-threatening hemorrhage [6]. The gold standard for diagnosing melioidosis-related mycotic aneurysms is a positive blood or tissue culture, combined with radiological evidence of arterial abnormalities in the abdomen and pelvis. These abnormalities are typically observed on CT imaging as multi-lobular or saccular aneurysms with a periaortic soft tissue mass [10].

Anunnatsiri et al. reported a high inpatient mortality rate of 20%, as many patients were admitted with signs of an impending aneurysm rupture [7]. Similarly, Wu et al. observed a mortality rate of 25% despite surgical intervention and appropriate antibiotic therapy. The clinical manifestations of mycotic aneurysms are often non-specific; among 77 cases, 56 patients presented with fever and chills, followed by abdominal and back pain [10]. In this case report, the patient presented with back pain, initially attributed to mechanical causes, leading to a delay in appropriate investigation. This highlights the critical importance of maintaining a high index of suspicion for melioidosis-related mycotic aneurysms in patients from endemic areas, particularly those with specific risk factors.

Early surgical intervention, such as urgent in-situ or extra-anatomical repair combined with debridement and appropriate antibiotic therapy, is the cornerstone of treatment for melioidosis-related mycotic aneurysms [10]. *B. pseudomallei* is intrinsically resistant to many antimicrobial agents. The recommended regimen includes intravenous ceftazidime or carbapenem for a minimum of six weeks during the intensive phase, followed by a trimethoprim/sulfamethoxazole-based eradication phase [11]. Oral doxycycline is an alternative for patients who are unable to tolerate trimethoprim/sulfamethoxazole. Despite appropriate treatment, the relapse rate is approximately 10%, increasing to nearly 30% with antibiotic durations shorter than eight weeks. Risk factors for relapse include non-adherence to treatment and the initial severity of the disease [12].

## Conclusions

This case underscores the importance of early diagnosis of melioidosis-related mycotic aneurysms and the need to consider empirical antimicrobial therapy in patients with risk factors for melioidosis. Timely surgical intervention, coupled with appropriate antibiotic use, is crucial for optimizing patient outcomes. Additionally, appropriate imaging should be conducted for patients with clinical suspicion of melioidosis-related mycotic aneurysms, particularly those presenting with back pain.

## References

[1] Wiersinga WJ, Virk HS, Torres AG, Currie BJ, Peacock SJ, Dance DA, Limmathurotsakul D (2018). Melioidosis. Nat Rev Dis Primers.

[2] Suputtamongkol Y, Chaowagul W, Chetchotisakd P (1999). Risk factors for melioidosis and bacteremic melioidosis. Clin Infect Dis.

[3] Chang CY, Lau NL, Currie BJ, Podin Y (2020). Disseminated melioidosis in early pregnancy - an unproven cause of foetal loss. BMC Infect Dis.

[4] Currie BJ, Mayo M, Ward LM (2021). The Darwin Prospective Melioidosis Study: a 30-year prospective, observational investigation. Lancet Infect Dis.

[5] Chang CY (2020). Periorbital cellulitis and eyelid abscess as ocular manifestations of melioidosis: a report of three cases in Sarawak, Malaysian Borneo. IDCases.

[6] Li PH, Chau CH, Wong PC (2015). Melioidosis mycotic aneurysm: an uncommon complication of an uncommon disease. Respir Med Case Rep.

[7] Anunnatsiri S, Chetchotisakd P, Kularbkaew C (2008). Mycotic aneurysm in Northeast Thailand: the importance of Burkholderia pseudomallei as a causative pathogen. Clin Infect Dis.

[8] Currie BJ, Ward L, Cheng AC (2010). The epidemiology and clinical spectrum of melioidosis: 540 cases from the 20 year Darwin prospective study. PLoS Negl Trop Dis.

[9] Tong TK, Shan G, Sibangun FJ, Keung BL (2021). Melioidosis-related mycotic aneurysm: three cases. IDCases.

[10] Wu H, Wang X, Zhou X, Wu Z, Wang Y, Pan M, Lu B (2020). Mycotic aneurysm secondary to melioidosis in China: a series of eight cases and a review of literature. PLoS Negl Trop Dis.

[11] Sullivan RP, Marshall CS, Anstey NM, Ward L, Currie BJ (2020). 2020 Review and revision of the 2015 Darwin melioidosis treatment guideline; paradigm drift not shift. PLoS Negl Trop Dis.

[12] White NJ (2003). Melioidosis. Lancet.

